# Age-shifting in malaria incidence as a result of induced immunological deficit: a simulation study

**DOI:** 10.1186/s12936-015-0805-1

**Published:** 2015-07-25

**Authors:** Peter Pemberton-Ross, Thomas A Smith, Eva Maria Hodel, Katherine Kay, Melissa A Penny

**Affiliations:** Swiss Tropical and Public Health Institute, 4002 Basel, Switzerland; Universität Basel, 4003 Basel, Switzerland; Liverpool School of Tropical Medicine, Liverpool, L3 5QA UK

**Keywords:** Malaria, Epidemiology, Vaccines, Chemoprevention, Age shift

## Abstract

Effective population-level interventions against *Plasmodium falciparum* malaria lead to age-shifts, delayed morbidity or rebounds in morbidity and mortality whenever they are deployed in ways that do not permanently interrupt transmission. When long-term intervention programmes target specific age-groups of human hosts, the age-specific morbidity rates ultimately adjust to new steady-states, but it is very difficult to study these rates and the temporal dynamics leading up to them empirically because the changes occur over very long time periods. This study investigates the age and magnitude of age- and time- shifting of incidence induced by either pre-erythrocytic vaccination (PEV) programmes or seasonal malaria chemo-prevention (SMC), using an ensemble of individual-based stochastic simulation models of *P. falciparum* dynamics. The models made various assumptions about immunity decay, transmission heterogeneity and were parameterized with data on both age-specific infection and disease incidence at different levels of exposure, on the durations of different stages of the parasite life-cycle and on human demography. Effects of transmission intensity, and of levels of access to malaria treatment were considered. While both PEV and SMC programmes are predicted to have overall strongly positive health effects, a shift of morbidity into older children is predicted to be induced by either programme if transmission levels remain static and not reduced by other interventions. Predicted shifting of burden continue into the second decade of the programme. Even if long-term surveillance is maintained it will be difficult to avoid mis-attribution of such long-term changes in age-specific morbidity patterns to other factors. Conversely, short-lived transient changes in incidence measured soon after introduction of a new intervention may give over-positive views of future impacts. Complementary intervention strategies could be designed to specifically protect those age-groups at risk from burden shift.

**Electronic supplementary material:**

The online version of this article (doi:10.1186/s12936-015-0805-1) contains supplementary material, which is available to authorized users.

## Background

Countries and organizations aiming to reduce the public-health burden of malaria have at their disposal an increasing number of options for intervention. These cover a wide range of modes of action, potential strategies of deployment and cost, making comprehensive analysis of effectiveness and cost-effectiveness challenging. It is not possible to cover all intervention combinations of interest by field trials, thus modeling and simulation can be useful to interpolate, optimize intervention packages and explore hypotheses [1].

This is particularly the case for analyses of the long-term effects of infection blocking interventions such as long-lasting insecticide-treated nets (LLINs) [2–4] or pre-erythrocytic vaccines (PEV) [5–7], but also to chemotherapy and prophylaxis strategies including test and treat [2], mass drug administration (MDA) [8] or seasonal malaria chemo-prevention (SMC; formerly referred to as Intermittent Preventive Treatment in children or IPTc) [9]. These interventions drive complex interplay of exposure and delay of natural immunity that may have counter-intuitive effects on subsequent transmission and burden [10, 11]. The advantages of infection blocking interventions are clear; the immediate burden associated with the infection is averted, and at the same time onward transmission of the parasite can be prevented with high enough coverage of the whole population, and thus not just those receiving the intervention may be protected [12].

Such interventions directly reduce immune challenge to individuals using the interventions. MDA strategies typically stipulate age-stratified cohorts for treatment, which even in the ideal scenario of a programme with perfect coverage and compliance will only cover a given individual for the length of time they remain in the designated age band [13]. Current strategies for interventions often target children under five years of age, leaving them without protection at older ages. Excess disease incidence resulting from the immunological opportunity cost will, therefore, be expected in age groups older than those treated if everything else remains static. In addition, reductions in transmission either to intervened and non-intervened individuals also reduce immune challenge and hence acquisition of natural immunity and also potentially allows decay of pre-existing immunity. This does not argue against interventions that delay immunity acquisition, but highlights the additional need to continue or increase coverage interventions for these individuals.

Averting infection and disease in one age group is thus likely to be accompanied by an excess of episodes in older age groups of the same individuals: referred to as an age-shift. Such age-shifts of clinical disease are a result of changes in exposure and delay of blood-stage malaria infection immunity acquisition, and are a general characteristic of infectious disease epidemiology [14], especially where (as with malaria) there is typically endemic stability [15] rather than epidemics. There have consequently been recurrent suggestions that interventions blocking malaria infection may lead to increases in clinical and severe disease burden in older individuals or at later time points [16–20]. While age-shifts, delays or rebounds are clearly predicted by many theoretical models of malaria dynamics [15, 21], direct measurement of these effects is generally impractical because of the short duration of most field trials, such as those used to establish the efficacy of ITNs [22–24] or of the RTS,S PEV [25, 26]. Where randomization is maintained for long enough, as in recent Phase II RTS,S trials [27, 28], a conventionally limited empirical analysis (i.e., one without accompanying serological data) cannot distinguish such shifts from the mechanistically distinct explanation of age- or time-dependence in the efficacy of the intervention. When a single population is followed up over a long period and effectiveness of interventions compared over time, (e.g. [29]) there is likely to be confusion about whether decreases in effect size are due to age-shifts and delayed morbidity or to resistance or insensitivity to the intervention (e.g. [30]).

Field studies of age- and exposure-dependent prevalence and the incidence of disease [31–33] can be used to indicate how the steady-state patterns will be modified by reductions in malaria transmission. In endemic areas where repeated infection occurs, naturally immunity is acquired which reduces the frequency of clinical episodes but does not reach the level of complete resistance. This partial immunity acts to reduce the parasite load in infected individuals and is a major determinant of malaria incidence patterns, providing some protection against severe morbidity and mortality in older children and adults, particularly in hyper- and holo-endemic areas [34]. The exact mechanisms of acquisition and decay of this immunity are poorly understood but the age- and exposure-relationships in the absence of intervention are well known. While protection against severe disease is developed after even small numbers of infectious mosquito bites [35], recurrent infections are needed for the host to become clinically immune, and even lifelong exposure does not lead to solid protection against infection. This has the consequence that areas with higher transmission intensities have lower risk of severe malaria after the first few years of life [36–38], and an earlier and narrower age-range of susceptibility to all disease [31].

The timing of age-shifts, delays, or rebounds depend on the transient dynamics of the system which in turn depend on the durations of the different stages of the parasite life-cycle and the dynamics of human demography [39]. These factors together determine how population immunity changes over time, and prediction of age-shifts needs to take them all into account. Field studies generally cannot directly estimate these effects, but by using simulation models which account for the complex dynamics of malaria transmission, immunity and morbidity that are parameterized with field data these effects and hypotheses can be explored. The magnitude of age-shifts induced by PEV and MDA have been simulated using an ensemble of open-source individual-based stochastic models of *P. falciparum* dynamics (*OpenMalaria*) that have been fitted to extensive data on age- and exposure-patterns of prevalence and disease [40, 41]. Using this framework we simulate the effect of the introduction of PEV and SMC programmes, using standard deployment strategies into endemic settings. We do this to look at predictions as a result of examples of induced immunological deficit. We assume transmission remains static for the period of follow-up, noting that in field studies this is likely not to be the case and thus the predicted magnitude of age-shifting is likely to be larger than in reality if there is increasing coverage of LLINs and access to treatment seen in recent years. Quantities of epidemiological interest were tracked as the programmes continued over a timespan of 20 years. The results are compared to outcomes in a control population that does not receive either intervention.

## Methods

*OpenMalaria* comprises an ensemble of discrete-time individual-based models of malaria in humans [40, 41] linked to population models of malaria in mosquitoes [42], and a dynamic model of human demography [43]. At each time-point the simulations contain a representation of the parasite densities of each *P. falciparum* infection in each human in the model, as well as the infectiousness to mosquitoes and morbidity status. The mosquito populations are classified into uninfected, infected and infectious vectors. Immunity is modelled in *OpenMalaria* via effects on parasite densities, which are reduced according to that individual’s history of infections, taking into account the previous total cumulative parasite load exposure and number of infection events. Several models in the ensemble allow for immunity decay. The details of these models and the fitting of the model parameters to data are outlined in [40, 41].

The *OpenMalaria* framework includes model components of not just transmission dynamics, human infection, immunity acquisition and decay, the life cycles of the mosquito and the malaria parasite, but also interventions aimed at different parts of the malaria life-cycle such as LLINs and the availability and efficacy of drugs and the health system. This allows for a wide range of malaria interventions to be simulated and investigated and impact on disease burden and transmission dynamics estimated. Malaria interventions are parameterized as much as possible using variables which correspond to real-world observables, to allowing one to simulate the effect of measurable changes in the local transmission environment. In particular, the ability to model vaccines and mass drug programmes at the population level and the pharmacokinetic (PK) profile at the individual level have made this investigations of both PEV and SMC feasible.

The ensemble of simulation models was previously described in detail by [41]. The present work uses a subset of six of the model variants listed in Table 2 of that [41]. The model variants are discrete time micro-simulations of malaria in humans, originally developed for modeling of malaria vaccines [40].

Models for case-management [44], PEV [45] and for clinical outcomes and mortality are described by [46, 47]. Each of these papers gives details of the rationale for the model structure. Model parameterisation is described by [41]. Implementation of these models is described at: https://github.com/SwissTPH/openmalaria/wiki.

For each simulation, the infection-blocking intervention (PEV or SMC) was introduced into a population of 100,000 people with endemic transmission with a seasonal pattern of two transmission seasons per year, similar to that found in certain sites in West Africa [41]. All individuals within the population were tracked for the first 20 years of the intervention, and yearly surveys were taken to record the age-stratified episodes of uncomplicated malaria, episodes of severe malaria, hospitalizations due to severe malaria and direct and indirect mortality. The age-groups for stratification are given in Table 1. Disability-adjusted life years (DALYs) were calculated from these indicators using the method presented in [43]. These indicators in the treated population were compared to a control cohort in a population that does not receive either intervention to provide a dynamic and age-specific picture of burden shift.

**Table 1:** Simulation parameters

Variable	Levels simulated for PEV and SMC	
Model variants	(1) R0000 base model	
	(2) R0068 heterogeneity in transmission: within-host variability	
	(3) R0131 immunity decay in effective cumulative exposure	
	(4) R0132 immunity decay in immune proxies	
	(5) R0133 immunity decay in both immune proxies & effective cumulative exposure	
	(6) R0670 heterogeneity in susceptibility to co-morbidity	
Population size	100,000	
Age-group upper bounds (years)	1, 2, 3, 4, 5, 6, 10, 12, 16, 20, 30, 40, 50, 60, 70, 99	
Survey intervals	Yearly surveys for 20 years	
Transmission pattern	Seasonal, West Africa	
EIR (infectious bites per person per year)	0.1^a^, 1, 2, 4, 8, 16, 64, 256	
Uncomplicated case management^b^ (%)	0, 5, 40	
Inpatient care for severe cases^c^ (%)	0, 100	
Vaccination coverage (%)	0, 100	

	PEV only	SMC only
Cohort age	EPI cohort 6, 10, 14 weeks old	All children aged between 3–59 months
Initial efficacy against infection (%)	62.7	100
Half-life (years)	1.12	0.175
Weibull decay shape parameter (*k*)	1 (exponential decay)	3.300 (slow decay, followed by quick decay)
Number of simulations	67,680	10,080

^a^EIR of 0.1 was not simulated, but any predictions for this level are taken as 10% of EIR 1.

^b^Probability of access to treatment for uncomplicated disease during a 5-day period.

^c^Probability of access to hospital care (or equivalent) for severe disease during any 5 day period.

To simulate both PEV and SMC simulations, six model variants from the *OpenMalaria* repertoire [41] (Penny MA, Galactionova K, Tarantino M, Tanner M, Smith TA: The public health impact of malaria vaccine RTS,S in malaria endemic Africa: country-specific predictions using 18 month follow-up Phase III data and simulation models. BMC Medicine, forthcoming) were employed, with each variant including the same sub-model for pathogenesis and case-management, but differing by assumptions concerning immunity decay or heterogeneity in transmission or co-morbidity. Each of these has been parameterized by fitting to observed relationships between seasonal patterns of EIR and a range of outcomes, including parasite prevalence and morbidity rates. This ensemble of models is used to provide insight into the scale of structural model error in the predictions. For each model variant a range of different EIRs and levels access to effective care was simulated, Table 1. The inclusion of effects of curative malaria treatment is a crucial part of our simulations. Effective treatment which clears the blood stage of the malaria parasite reduces the length of a given malaria episode, and the chance of recurrences, severe sequelae and death. This has downstream impacts on the future immunity state and infectiousness of individuals, and the population-wide prevalence and transmission dynamics which feedback in a complex nonlinear manner. In this study, it was assumed that each individual had a $14.5\%$ probability of receiving blood-stage clearance of the malaria parasite in any two week period with illness (intended to correspond to the situation in some areas of Senegal [48]). This summary probability thus takes into account access to treatment, treatment adherence and compliance and any drug resistance.

The simulation of vaccination comprised the administration of a PEV of three doses according to the schedule of the WHO Expanded Program on Immunization (EPI), with $93\%$ coverage at third dose achieved at an age of 14 weeks in the cohort, based on data from Penny MA, Galactionova K, Tarantino M, Tanner M, Smith TA (The public health impact of malaria vaccine RTS,S in malaria endemic Africa: country-specific predictions using 18 month follow-up Phase III data and simulation models. BMC Medicine, forthcoming). The simulated vaccine confers protection in this age group of initial efficacy against infection of $62.7\%$ at third dose, which decays exponentially with a half-life of 1.12 years (Penny MA, Galactionova K, Tarantino M, Tanner M, Smith TA: The public health impact of malaria vaccine RTS,S in malaria endemic Africa: country-specific predictions using 18 month follow-up Phase III data and simulation models. BMC Medicine, forthcoming). Effects of incomplete courses of vaccination were not included. The quoted results are derived as weighted averages of outputs for the different vaccine profiles detailed in Table 1 and methodology detailed in Penny MA, Galactionova K, Tarantino M, Tanner M, Smith TA (The public health impact of malaria vaccine RTS,S in malaria endemic Africa: country-specific predictions using 18 month follow-up Phase III data and simulation models. BMC Medicine, forthcoming).

**Figure 1:**
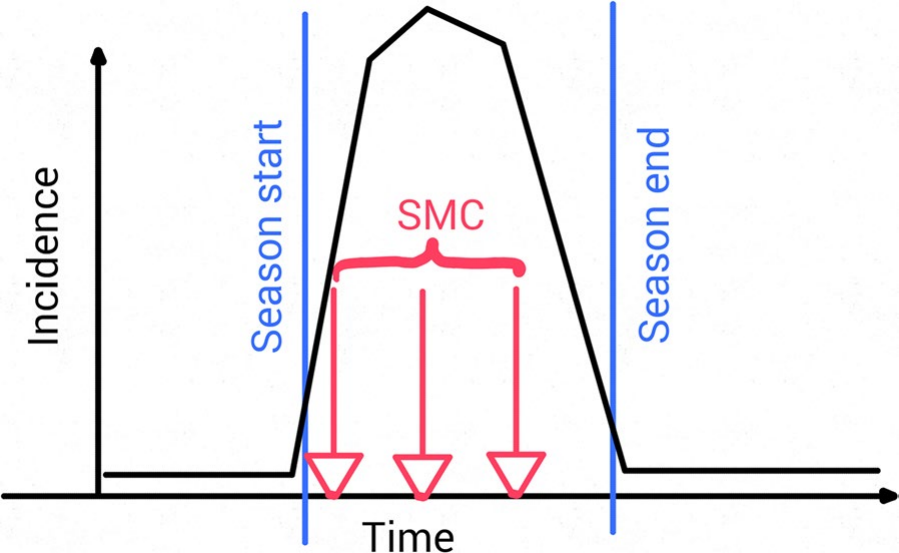
Up to four doses are provided every season at monthly intervals during the main transmission season, according to the WHO-MPAC recommendation.

The SMC programme was implemented in the simulations similar to that recommended by the WHO-Malaria Policy and Advisory Committee (MPAC) [49]. The simulated pharmacodynamics (PD) were based on the effects of a combination of amodiaquine (AQ) and sulphadoxine–pyrimethamine (SP) administered monthly to all children aged 3–59 months during the transmission season, with a maximum of four administrations per season, Figure 1. Simulations of AQ-SP drug action were based on two previously calibrated and validated pharmacological models of artesunate-AQ [50] and SP [51] treatment. The PK model for AQ assumed first-order absorption, linear elimination and used a pair of two-compartment disposition models to track the drug concentration of both the parent drug and active metabolite, in parallel over time [52]. This was combined with a PD model following Michaelis–Menton kinetics as described in [50]. The PK model for SP assumed both drugs were instantaneous absorbed, followed one-compartment kinetics and had linear elimination [51]. Sulphadoxine and pyrimethamine act synergistically in combination and so drug effect was determined from an isobologram describing parasite survival in the presence of various SP concentrations [53] as described in [51]. The prophylactic effect $\epsilon (t)$ of this combination against re-infection over time *t* was parameterised by simulating amodiaquine and sulphadoxine–pyrimethamine administered to 1,000 individuals and estimating the probability infections were prevented in time for the appropriate age groups. This was fit to a Weibull decay curve of the form

1 $$\begin{aligned} \epsilon (t) = \exp \left( -\left( \frac{t}{\lambda }\right) ^k \log 2 \right) \end{aligned}$$

with half-life $\lambda = 0.175$ years and shape parameter $k = 3.300$. The quoted results are derived as weighted averages of outputs for different vaccine profiles as detailed in Penny MA, Galactionova K, Tarantino M, Tanner M, Smith TA (The public health impact of malaria vaccine RTS,S in malaria endemic Africa: country-specific predictions using 18 month follow-up Phase III data and simulation models. BMC Medicine, forthcoming).

Model averaging was used to provide a representative picture of the typical observed dynamics over the a range of values for each of the factor levels simulated listed in Table 1, as in Penny MA, Galactionova K, Tarantino M, Tanner M, Smith TA (The public health impact of malaria vaccine RTS,S in malaria endemic Africa: country-specific predictions using 18 month follow-up Phase III data and simulation models. BMC Medicine, forthcoming). The PEV simulations were weighted to simulate access to care, vaccination coverage and transmission profiles comparable to those found in areas in West Africa. The weights used for transmission level were computed based on the prevalence rasters in [54], which were transformed into EIR values using the relationship in Penny MA, Maire N, Bever C, Pemberton-Ross P, Briët OJT, Smith DL, et al. (Distributions of malaria exposure in endemic countries in Africa considering country levels of effective treatment, submitted) and the estimated level of access to effective care. This level was scaled from Demographic and Health Surveys (DHS) at admin-1 level [48]. The predictions of the SMC programme in the population are derived as weighted averages of outputs as detailed in Penny MA, Galactionova K, Tarantino M, Tanner M, Smith TA (The public health impact of malaria vaccine RTS,S in malaria endemic Africa: country-specific predictions using 18 month follow-up Phase III data and simulation models. BMC Medicine, forthcoming).

## Results

**Figure 2:**
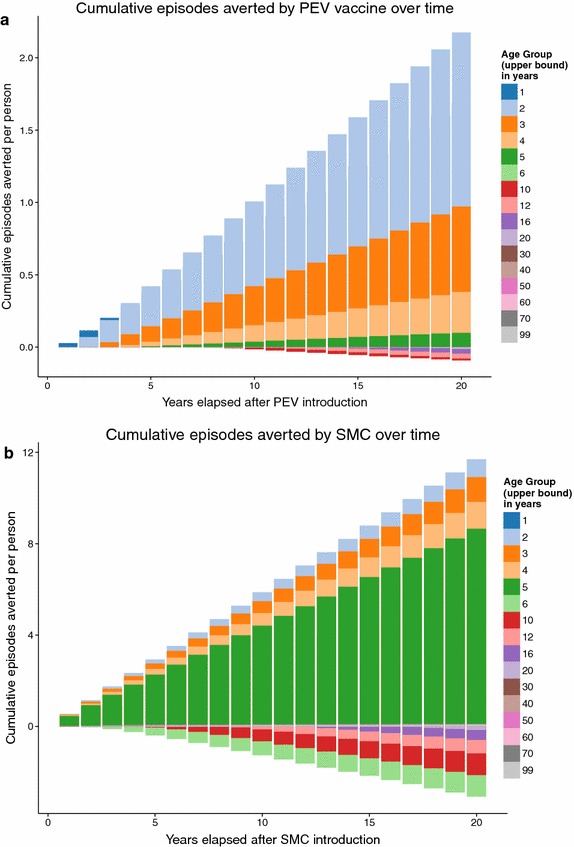
Cumulative uncomplicated episodes averted over time. The age-groups affected by burden shift differ markedly between PEV and SMC programmes.

**Figure 3:**
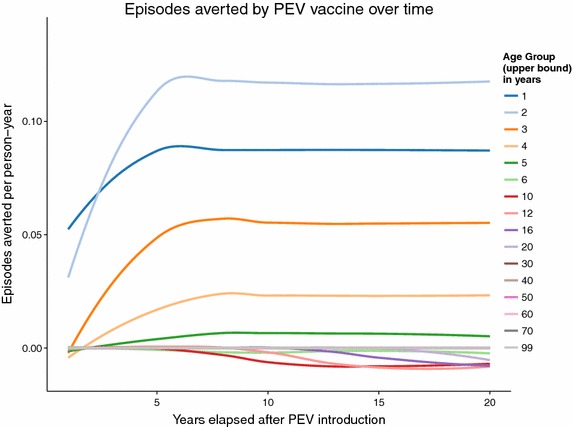
Averted episodes of uncomplicated malaria in each age group per person-year, in each year of the 20 year course of the PEV programme.

**Figure 4:**
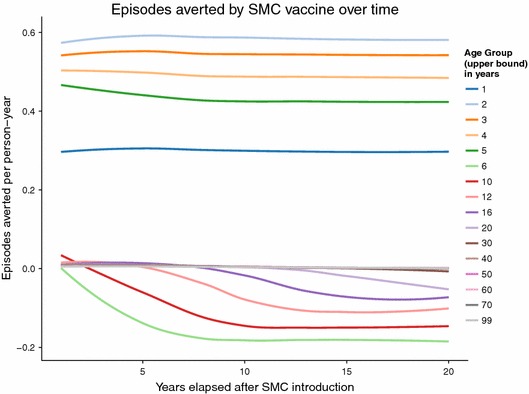
Averted episodes of uncomplicated malaria in each age group per person-year, in each year of the 20 year course of the SMC programme.

**Figure 5:**
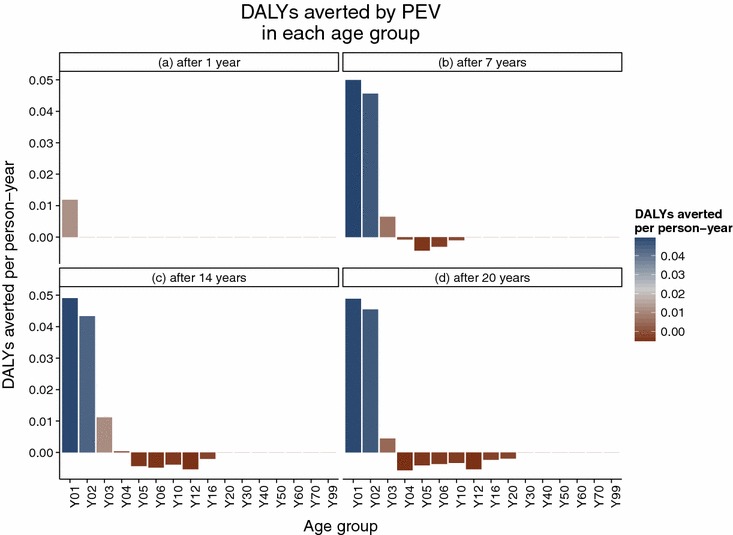
Averted DALYs at four timepoints in the PEV programme.

**Figure 6:**
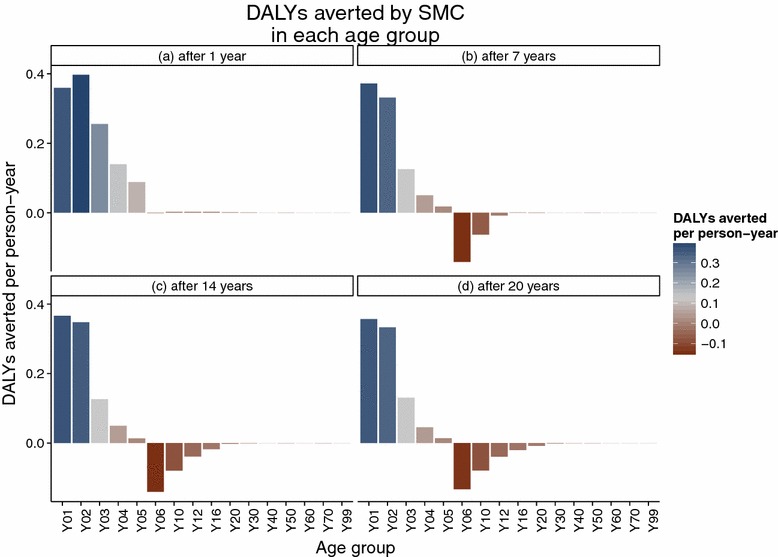
Averted DALYs at four timepoints in the SMC programme.

The simulated SMC programme assumes frequent treatment, resulting in predictions of much larger numbers of episodes averted than the PEV programme Figure 2, but with both programmes, over the whole of the 20 year follow-up period, the average predicted number of malaria episodes show an excess in certain age groups compared to the control untreated cohort. In the PEV simulation, episodes of averted uncomplicated disease are predicted in the five youngest age groups (0–1, 1–2, 2–3, 3–4 and 4–5 year olds) in all years of the programme (Figure 3), but this is accompanied by an excess of episodes predicted in all age groups between 5 and 20 years of age. No effect in the age groups older than 20 years old is observed in the predictions due to the 20 year time span of the simulation follow-up, and the lack of appreciable predicted population effect on transmission of simulated PEV introduced via EPI [5]. The first observed onset of excess episodes for the 5–6 year old age group occurs as early as 4–5 years after the start of vaccination, the earliest time point a vaccinated child would reach this age. A similar temporal pattern to the onset of excess uncomplicated cases is predicted for the SMC programme (Figure 4) although this intervention affects older age groups than the PEV programme and is thus accompanied by a relatively quicker onset and greater number of averted cases in the treated age groups, particularly in the 4–5 year old age group. The age and time pattern of clinical cases averted, and subsequent excess of cases in the intervened individuals, is also predicted for the averted DALYs distribution over time, Figures 5 and 6. After the initial year of the PEV programme, Figure 5, DALYs are averted constantly in the 0–1 and 1–2 year old age groups. However, as the study progresses excess DALYs are seen in an increasing number of age groups, as more age cohorts comprise previously vaccinated individuals with reduced natural immunity compared to same age control cohorts. The peak of the distribution of excess DALYs remains constant around the 4–5 and 5–6 year old age groups. A similar pattern of excess cases and DALYs are seen in the SMC programme, Figure 6, affecting older age groups than the PEV due to the older ages of intervention coverage. Averted DALYs in the youngest age groups are predicted in the first year of the SMC programme, much sooner than in the PEV programme.

**Figure 7:**
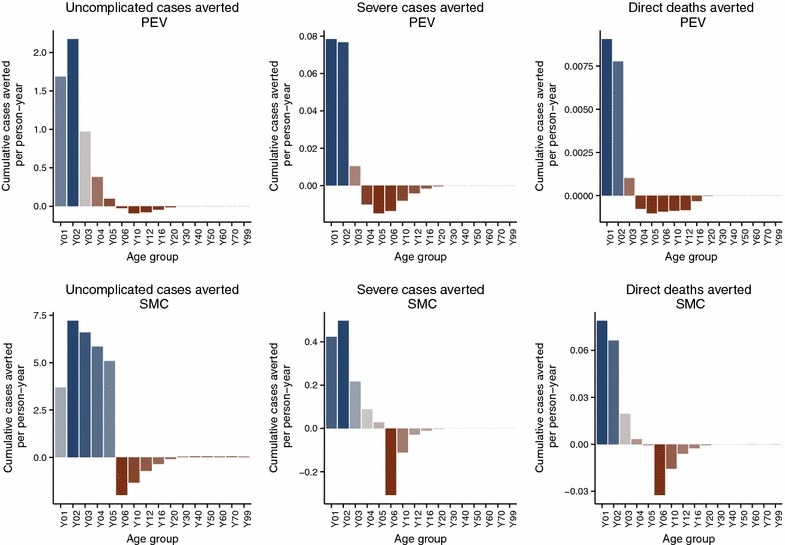
Cumulative averted episodes in each age group per person-year, over the 20 year course of both PEV and SMC programmes.

Predictions of excess uncomplicated episodes in the age groups older than 5 years old accumulate over time in both PEV and SMC programmes, Figure 7. Cumulative excess severe disease and deaths directly attributable to malaria are seen in all ages $\ge$4 years old for PEV and $\ge$6 years old for SMC, the difference between interventions attributable to the continued administration of SMC to 2–5 year olds.

**Figure 8:**
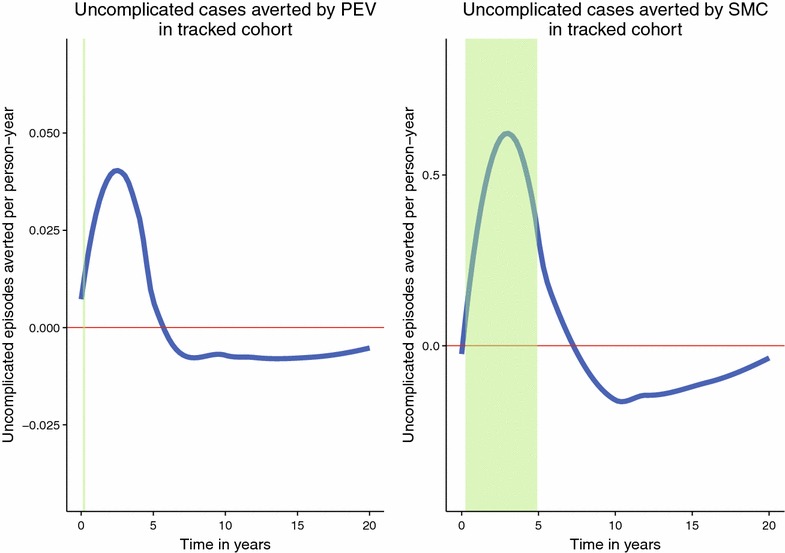
Uncomplicated episodes averted in cohort born in the first year of the programmes. The* areas shaded green* indicate the ages at which the intervention is administered. The burden averted in the first 5 years of life is partially shifted to later life, particularly the 5 years immediately following. Although the scale of the effects differs markedly between the two interventions, the general profile is quite similar.

The same effect is predicted if we track a cohort through time, Figure 8. Individuals born in the first year of the simulation exhibit a similar pattern of averted uncomplicated episodes, with up to 0.6 episodes per person year averted by SMC at the age of 3, accompanied by an excess of up to 0.2 episodes per year at the age of 9. A similar dynamic is observed for the PEV, Figure 8. Burden is partially shifted from the first 5 years of life primarily to the following 5 years, and excess burden is observed even at the age of 20 years old (i.e. as long as 14 years after the last administration of SMC).

**Figure 9:**
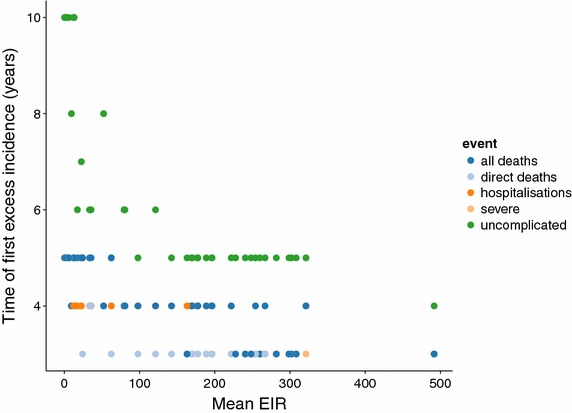
Time to first onset of excess incidence at a range of exposure levels in the PEV programme, coloured by episode severity. Multiple points at the same EIR represent simulations with different transmission models and random seed.

Excess severe disease and deaths are also predicted to occur earlier than excess uncomplicated disease, Figure 9, in some cases after little more than half the time after the start of the programme. For all episode severities, the first onset of excess cases happens sooner at high exposure, measured here by the entomological inoculation rate (EIR). At very low exposure (EIR = 1 infective bite per person per annum), the first onset of excess uncomplicated disease can be as late as 10 years after the start of the programme.

The predicted total number of uncomplicated episodes averted over the entire population is positive for both strategies, demonstrating, that despite predictions of age-shift in disease, there is an overall positive benefit of the programmes with more cases averted in the younger age groups than any possible predicted excess of cases when PEV protection wanes or children reach ages older than target SMC ages. The benefits are primarily predicted to be younger age-groups for PEV than for SMC, Figure 2. This is due to the target age groups of the interventions but also to the very large amount of disease averted in the intervened age groups immediately after the start of each programme.

**Figure 10:**
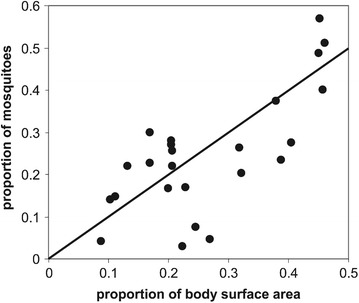
The effect of body surface area on mosquito bites received. The proportion of bites from* Anopheles gambiae* s.s mosquitoes received by the child is plotted against the proportion of the surface area contributed by the child. The* diagonal line* corresponds to direct proportionality.

**Figure 11:**
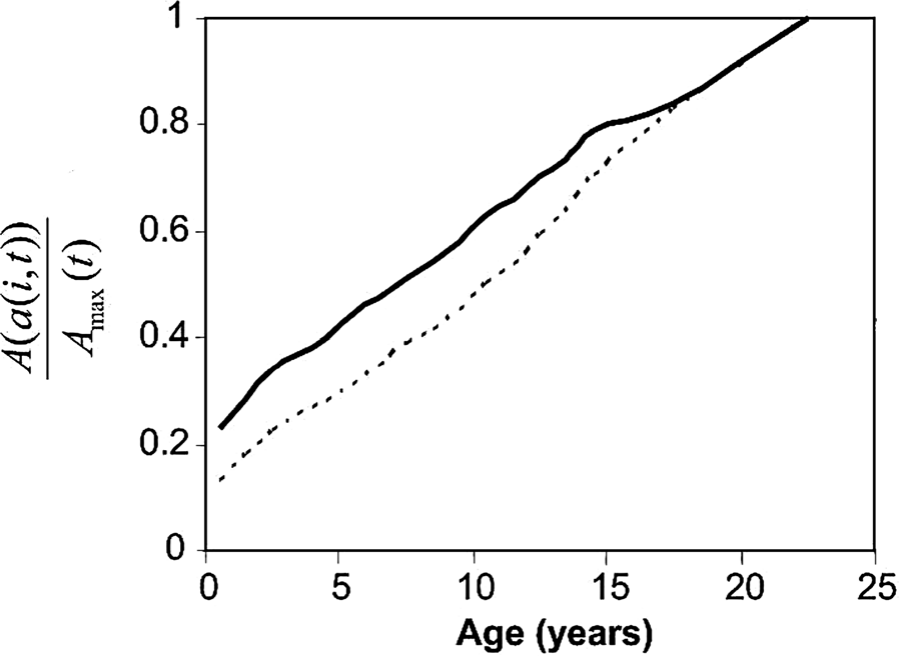
*OpenMalaria* function modelling the effect of age on number of mosquito bites received. Shown is the ratio of bites received to those received by an adult $(A(a(i,t))/A_{max}(t)$ by age *a*(*i*, *t*). The* continuous line* corresponds to proportionality between bites received and expected body surface area. The* dotted line* shows a similar model which assumes proportionality between bites received and body weight.

## Conclusion

Age-shifting of disease, delayed morbidity or rebounds in morbidity and mortality will generally occur when partially effective malaria interventions are deployed in ways that do not permanently interrupt transmission. Such age-shifts have been observed in previous field studies of chemoprophylaxis [20] and discussed at length in historical literature, but such effects will be hard to detect in field trials if follow-up is not long enough or background transmission decreases during trials. It is conjectured that all models of malaria dynamics that capture age patterns of transmission and disease incidence relationships for *P. falciparum* will predict these effects of age-shifting for any transmission reducing intervention that prevent infections in individuals, especially when targeted to young ages. The two malaria interventions considered here, PEV and SMC, covered different age groups for implementation, with both preventing new infections in individuals for varying lengths of time and in the case of SMC cleared infections when given. Both interventions are predicted to result in age-shifting of disease, with some excess clinical cases in older ages once the intervention no longer protects. Despite this age-shifting of disease, an overall positive benefit is predicted, with the number of clinical cases averted larger than any possible excess in older ages. This is in part due to the interventions targeting young ages where disease burden is higher. In simulations of naive and near naive individuals in settings with low malaria prevalence, the *OpenMalaria* models predict recurrent illness from single infections; they incorporate dynamic effects of treatment; and they reproduce the age patterns of uncomplicated episodes in a study [31] in which there is a strong ’cross-over’ effect in the sense that in older age groups, clinical incidence in the lower transmission site was higher than at higher exposure. In the models, these patterns are partly driven by the high treatment rates in these villages, a situation somewhat similar to an SMC programme. Other models might reproduce these effects to lesser extents if they are parameterised using different constraints and data [15].

Three different sets of scenarios leading to rebounds or age-shifts can be distinguished: (a) when intervention deployment over the whole population is maintained long-term at a constant level, there is typically an initial phase during which the burden reduction is maximal, followed by readjustment to a new steady-state during which there may be a temporary increases of morbidity rates above the steady-state levels. This pattern is clearly seen in *OpenMalaria* analyses of rebounds in long-term LLIN programmes [3]. (b) Discontinuation of programmes, such as single-round LLIN distributions [3], or weakening or cessation of sets of repeated programmes [55], is followed ultimately by a return to the original steady-state (assuming exogenous factors remain the same). The transient dynamics that may involve temporary increases of morbidity rates above the initial level. Situation (c), analysed in this paper, occurs when the intervention is provided only to a specific age range, but is maintained long-term. This shares some of the characteristics of (a) in that it potentially leads to a new steady state if the interventions reduce transmission, but is similar to (b), in that since the intervention is only applied to each individual for a finite period so each individual emerges from the protected age range with diminished immune readiness relative to non-intervened individuals and that expected for a given environment’s intrinsic potential for malaria. So the new steady state entails an age-shift in the pattern of disease. The timing of the effects for PEV and SMC are remarkably similar, though the ages affected are very different.

A general characteristic of all of these phenomena is that they are evident much sooner in high transmission settings, and that the largest burden shifts are also seen at the highest transmission levels. In such settings, one factor influencing burden shift to the 4–10 month age group is maternal immunity, as this is the age at which the protection conferred by transplacental maternal antibodies is expected to wane. This has the potential to leave this age group particularly vulnerable, as both maternal and clinical immunity diminish without the interim immune challenge required to produce a replacement protection [32]. However, this is likely to be an important factor only in very high transmission settings where a substantial amount of exposure is experienced in this short age-window.

In low-transmission settings, without increasing coverage of transmission reducing interventions, resurgence of disease may be distributed over a very long period after programme start, predicted to extend at least into the second decade. This makes it unlikely that this will be detected in the majority of controlled field trials of MDA or vaccination, which have relatively short follow-up periods. Many other changes in both malaria interventions and in environmental drivers of transmission are likely over such time-scales, so even if long-term surveillance is maintained it will be difficult to avoid mis-attribution of such long-term changes in age-specific morbidity patterns to other factors. Conversely, short-lived transient changes in incidence measured soon after introduction of a new intervention may give misleading views of future impacts [3]. An immediate drop in incidence in one age group may be interpreted as evidence of success, but this should always be judged in the context of longer time shifts in incidence in this and other age groups.

A key determinant of these long-term dynamics is the very slow decay of population immunity when the challenge is reduced. The analysis suggests that the immunological “opportunity cost” that is paid during the period in which new infections are blocked, mainly results from the recruitment of unexposed infants into the population, not from loss of immunological memory in previously exposed cohorts. While there is field evidence that treating asymptomatic infections increases subsequent susceptibility in the short-term [56–58], significant immunity persists for a very long time [59]. Only by assuming very slow decay of immunity (or none at all) could the *OpenMalaria* models be fitted to the available data [41, 43]. It follows that the timing and extent of age-shifts is sensitive to the birth-rate and age-distribution of the human population, and that models need to use realistic age-distributions if they are to give reliable indications of the extent of age-shifts.

The dynamics of age-shifts also depend on the slow build-up of natural immunity under parasitological challenge. An important feature of this is an increase in the force of infection with age for the first few years of life [60]. The *OpenMalaria* models capture this by assuming natural exposure is a function of body size, reflecting increasing biting by mosquitoes on larger hosts [61, 62] Figure 10 (figure taken from [63]). This is implemented as an age-dependent scaling factor, which expresses the number of bites received as a child as a fraction of the bites received by a fully-grown adult, proportional to the ratio in surface areas Figure 11 [63]. The consequent effect of child growth on exposure magnifies the age-shifting effect because when the exogenous protection of the treated cohort is lost at a time when their increased size leaves them more vulnerable than younger children.

Even sustained interventions which include more age-groups over longer periods will not necessarily entirely prevent age-shifting of disease [3], but there may be a benefit to the use of complementary interventions targeted specifically to those age-groups at risk of burden shift. This could take the form of further infection-blocking interventions such as increasing usage of LLINs in those age groups (with LLINS also reducing transmission by their direct effects on mosquitos) and PEV booster programmes, particularly if longer half-life PEVs become available. Further simulation studies would be useful to help optimise design of such mitigation strategies and investigate cost-effectiveness. The simulation predictions that, SMC averts clinical cases in the age group into which PEV induces excess cases suggests that SMC may be an appropriate strategy for mitigating the age shifts induced by a PEV, and will be a subject of future work. Modelling is useful to establish the optimal timing, cohorts and administration regimes for such combination programmes and would additionally provide cost-effectiveness estimates of intended programmes to national malaria control programs for budget impact analysis.

The age-patterns of infection and disease in the models were fitted to the data of a number of field studies (see Additional file 1), and the results are thus heavily driven by data, rather than by somewhat uncertain assumptions about pathogenesis and immunity. Empirical studies that have investigated age shifts of malaria interventions have found results similar to our simulations [20, 64]. However, generalization from models fitted to a limited set of locations is inevitably associated with uncertainties, especially since the age patterns of disease must depend on local factors such as patterns of co-morbidity. The greatest uncertainties attach to the assumptions about the models of intervention effects, especially of SMC, where the assumptions about the relationship between coverage and effective protection remain untested.

## Additional file

Additional file 1. Details of model variants used and SMC simulation results by model variant.
