# Supplementary material for: Age-shifting in malaria incidence as a result of induced immunological deficit: a simulation study
Source: Malar J. 2015 Jul 25;14:287. doi: 10.1186/s12936-015-0805-1 (PMC4513612; doi:10.1186/s12936-015-0805-1)
Supplement: Additional file 1. — Details of model variants used and SMC simulation results by model variant. [file 12936_2015_805_MOESM1_ESM.pdf]

Supplementary figures to Age-shifting in malaria incidence as a result of induced immunological deficit : a simulation study

July 8, 2015

Seasonal malaria chemoprohylaxis health outcomes, reported by model

The following figures demonstrate that the six *OpenMalaria* model variants used in this study make very similar predictions for disease outcomes. All six models display similar behaviour in their predictions of the temporal variations in events averted, Figures 1–3, and in their predictions of the affected age groups, Figures 4–6. As these are the primary inputs to our downstream analyses, e.g. the calculation of DALYs averted, these predictions will also be similarly uniform across the six models used.

Detailed lists of the parameters used in each model can be found in [1].

Author details

References

1. Smith T, Ross A, Maire N, Chitnis N, Studer A, Hardy D, et al. Ensemble modeling of the likely public health impact of a pre-erythrocytic malaria vaccine. PLoSMed. 2012 Jan;9(1):e1001157.

Table 1: **Simulation parameters**

Model	Description
R0000	Base model
R0068	Heterogeneity in transmission: within-host variability
R0131	Immunity decay in effective cumulative exposure
R0132	Immunity decay in immune proxies
R0133	Immunity decay in both immune proxies & effective cumulative exposure
R0670	Heterogeneity in susceptibility to co-morbidity

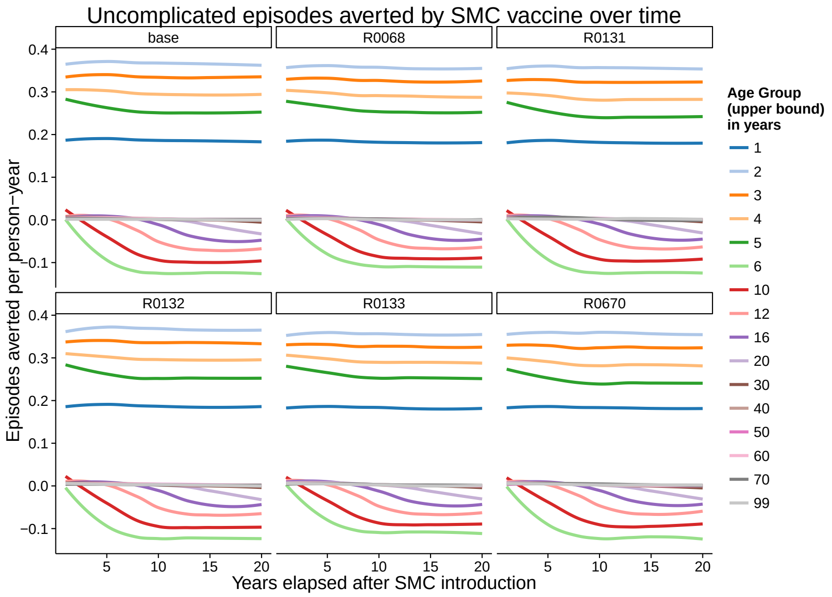

Figure 1: Uncomplicated episodes averted over time in each of the six models in the ensemble

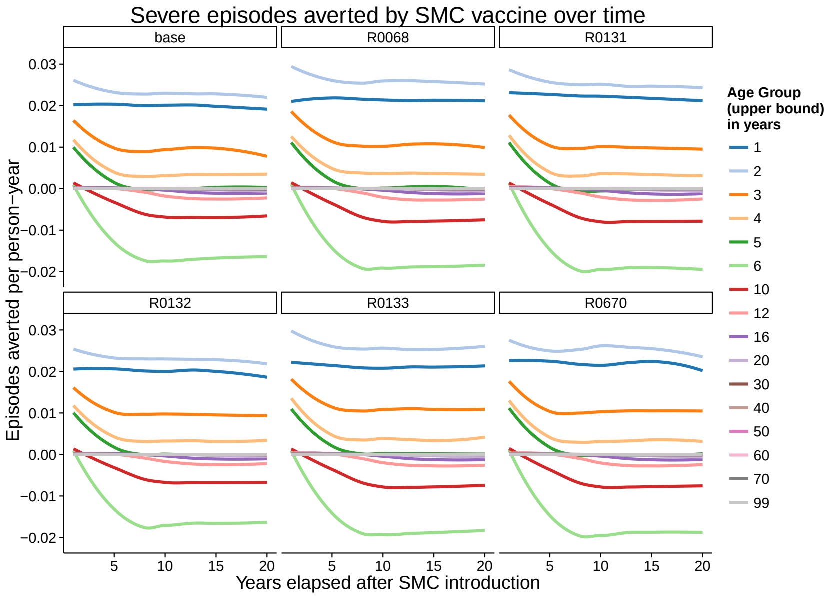

Figure 2: Severe episodes averted over time in each of the six models in the ensemble

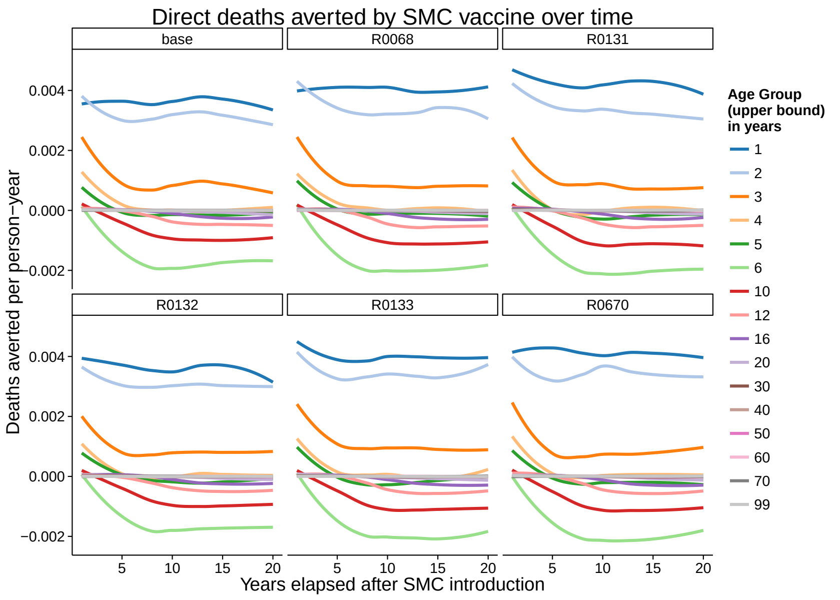

Figure 3: Direct deaths averted over time in each of the six models in the ensemble

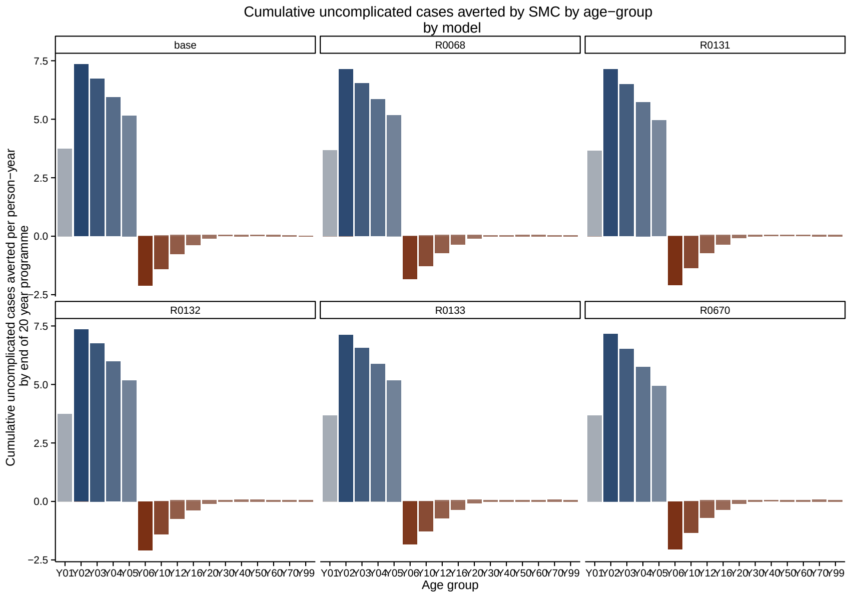

Figure 4: Cumulative uncomplicated episodes averted in each age group after 20 years in each of the six models in the ensemble

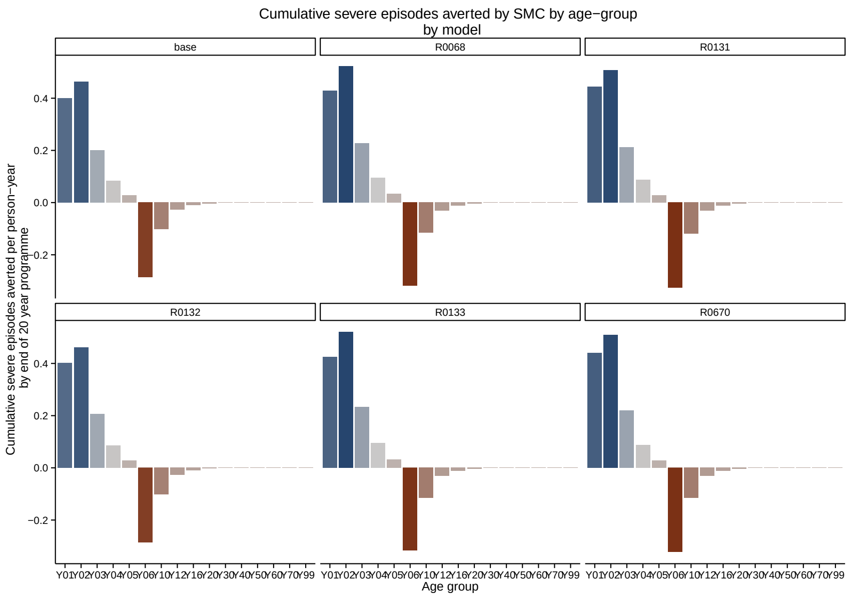

Figure 5: Cumulative severe episodes averted in each age group after 20 years in each of the six models in the ensemble

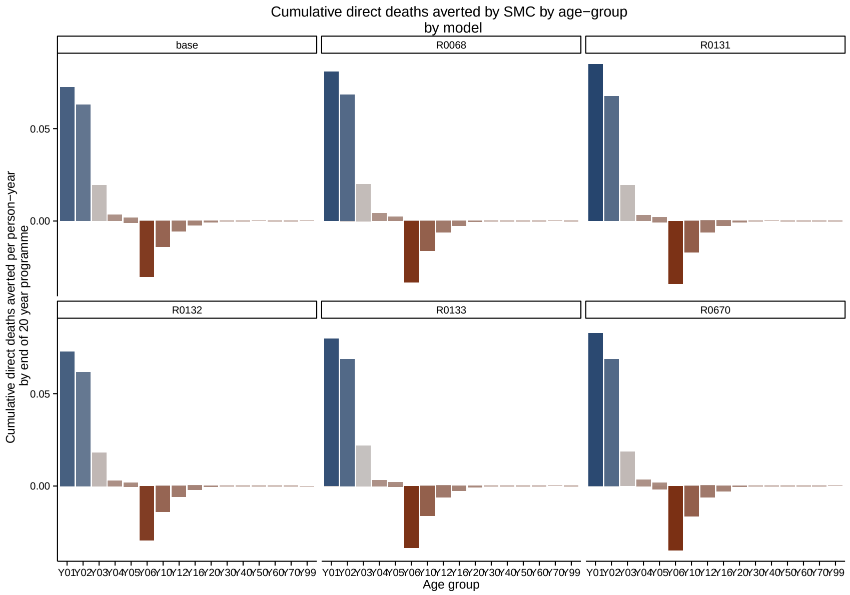

Figure 6: Cumulative direct deaths averted in each age group after 20 years in each of the six models in the ensemble
